# Unusual electronic and vibrational properties in the colossal thermopower material FeSb_2_

**DOI:** 10.1038/s41598-018-29909-2

**Published:** 2018-08-03

**Authors:** C. C. Homes, Q. Du, C. Petrovic, W. H. Brito, S. Choi, G. Kotliar

**Affiliations:** 1 Condensed Matter Physics and Materials Science Division, Brookhaven National Laboratory, Upton, New York, 11973 USA; 2 Department of Materials Science and Chemical Engineering, Stony Brook University, Stony Brook, New York, 11790 USA; 3 Department of Physics and Astronomy, Rutgers, The State University of New Jersey, Piscataway, New Jersey 08854 USA

## Abstract

The iron antimonide FeSb_2_ possesses an extraordinarily high thermoelectric power factor at low temperature, making it a leading candidate for cryogenic thermoelectric cooling devices. However, the origin of this unusual behavior is controversial, having been variously attributed to electronic correlations as well as the phonon-drag effect. The optical properties of a material provide information on both the electronic and vibrational properties. The optical conductivity reveals an anisotropic response at room temperature; the low-frequency optical conductivity decreases rapidly with temperature, signalling a metal-insulator transition. One-dimensional semiconducting behavior is observed along the *b* axis at low temperature, in agreement with first-principle calculations. The infrared-active lattice vibrations are also symmetric and extremely narrow, indicating long phonon relaxation times and a lack of electron-phonon coupling. Surprisingly, there are more lattice modes along the *a* axis than are predicted from group theory; several of these modes undergo significant changes below about 100 K, hinting at a weak structural distortion or phase transition. While the extremely narrow phonon line shapes favor the phonon-drag effect, the one-dimensional behavior of this system at low temperature may also contribute to the extraordinarily high thermopower observed in this material.

## Introduction

**Figure 1:**
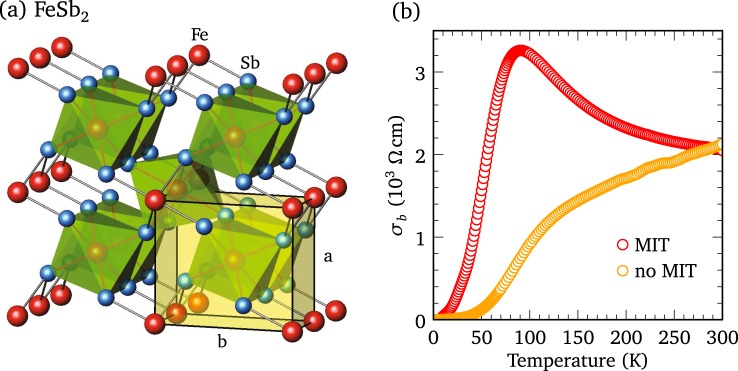
Structural and transport properties of FeSb_2_. (**a**) The crystal structure of FeSb_2_ in the orthorhombic *Pnnm* (58) setting is shown for an *a* *–* *b* face, with the *c* axis facing into the paper; there are two formula units per unit cell. The orthorhombic unit cell dimensions are roughly 5.83, 6.53 and 3.20 Å for the *a*, *b*, and *c* axis, respectively^35^. The fractional coordinates are Fe (0, 0, 0) and Sb (*x*, *y*, 0), with *x* = 0.1885 and *y* = 0.3561. Each Fe atom sits at the center of a deformed octahedra which share edges along the *c* axis. (**b**) The dc conductivity along the *b* axis, determined from the dc resistivity *σ*_*b*_ = 1/*ρ*_*b*_, is shown for the crystal that displays a MIT, and one that does not. The dc transport properties have been measured on the same samples that the optical measurements were performed.

FeSb_2_ crystallizes into an orthorhombic structure with two formula units per unit cell, as shown in Fig. 1(a). Despite this simple structure, there are two moieties of FeSb_2_ crystals, those with a putative metal-insulator transition (MIT) in which the dc conductivity along the *b* axis first increases below room temperature, reaching a broad maximum at about 80–100 K, before decreasing dramatically as the temperature is further reduced^1^, and a second class of materials without a MIT in which the dc conductivity immediately begins to decrease as the temperature is lowered^1–4^, as shown in Fig. 1(b), for the two types of crystals examined in this work. Both classes of materials have a high thermoelectric power factor at low temperature; however, it is extraordinarily high in the materials with a MIT^1^. The thermoelectric efficiency is given by the dimensionless figure of merit *ZT* = *σS*^2^*T*/*κ*, where *σ*, *S*, *T*, and *κ* are the conductivity, Seebeck coefficient, temperature, and thermal conductivity, respectively; the thermoelectric power is simply *S*^2^*σ*; in FeSb_2_ the Seebeck coefficient may be as high as $S\simeq -\,45$ mV K^−1^ at low temperature, resulting in the highest power factor ever recorded^2^. In general, there are two strategies for increasing *ZT*; reduce *κ* or increase the power factor *S*^2^*σ*. However, because the source of this large thermoelectric response is not entirely understood, with electronic correlations^1–11^, as well as the phonon-drag effect^11–15^, having been proposed, it is not clear which approach offers the best chance of success. The complex optical properties yield information about both the electronic and vibrational properties of a material, and can offer insights into the origin this unusual behavior. The real part of the optical conductivity is particularly useful as it yields information about the gapping of the spectrum of excitations in systems with a MIT, and in the zero-frequency limit, the dc conductivity is recovered, *σ*_1_(*ω* → 0) ≡ *σ*_d*c*_, allowing comparisons to be made with transport data. Furthermore, the infrared-active transverse-optic modes at the center of the Brillouin zone may be observed in *σ*_1_(*ω*) as resonances superimposed upon an electronic background (or antiresonances if strong electron-phonon coupling is present). The optical properties of FeSb_2_ have been previously examined in the *a-b* planes^6^ and along the *c* axis^7^, revealing a semiconducting response at low temperature and evidence for electron-phonon coupling.

## Results

Crystals of FeSb_2_ have been prepared by the usual methods^16,17^. The reflectance of several single crystals, with and without a MIT, has been measured over a wide frequency range ($\simeq 3$ meV to 4 eV) at a variety of temperatures for light polarized along the *a*, *b*, and *c* axes^18^ (Supplementary Fig. S1). Only naturally-occurring crystal faces have been examined, although after an initial measurement the *c* axis face was polished to remove some surface irregularities. Polishing broadens the lattice mode(s), but does not otherwise affect the optical properties. After the optical measurements were completed, the samples were dismounted and the dc resistivity, *ρ*_*dc*_, was measured using a standard four-probe technique^1^ [the dc conductivity, ${\sigma }_{dc}=\mathrm{1/}{\rho }_{dc}$, is shown along the *b* axis in Fig. 1(b)].

**Figure 2:**
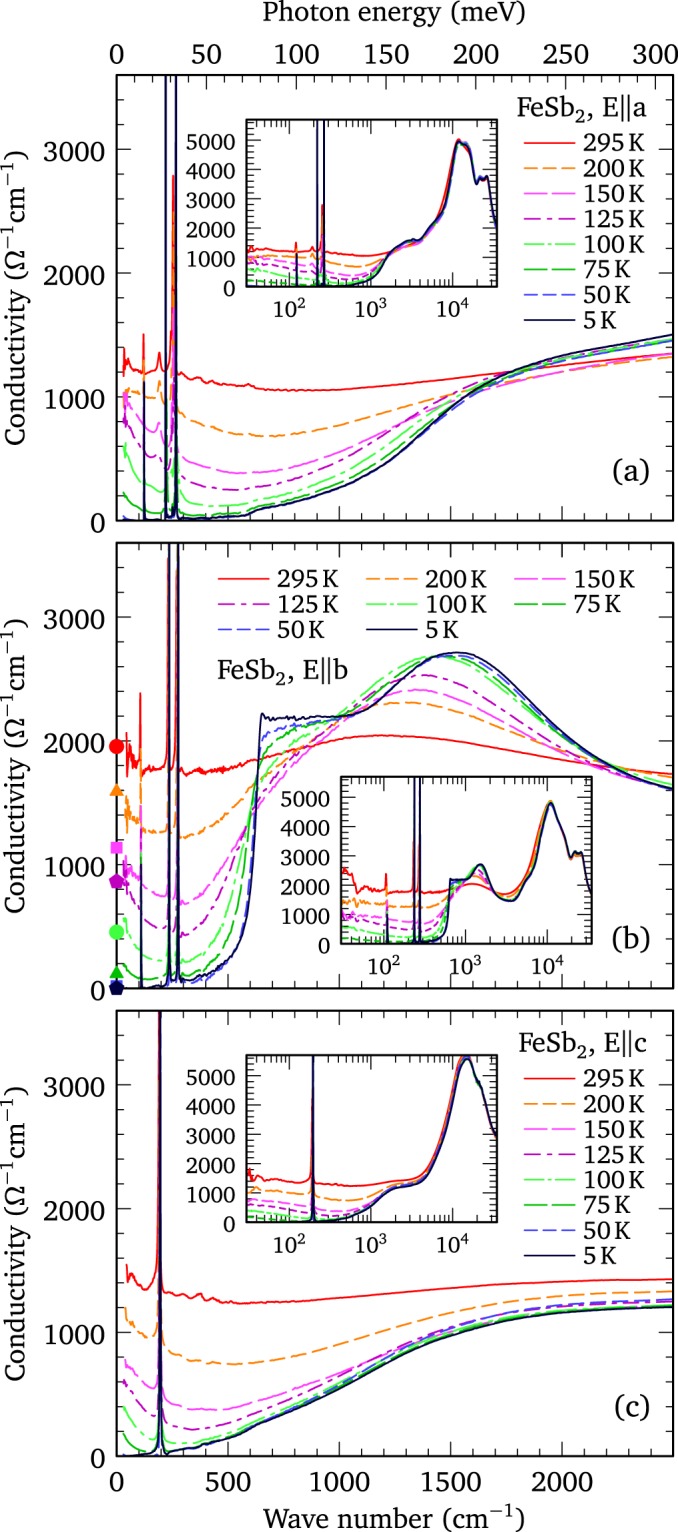
The optical conductivity of FeSb_2_. (**a**) The temperature dependence of the real part of the optical conductivity for light polarized along the *a* axis, revealing several extremely sharp infrared-active lattice modes and the rapid suppression of the low-frequency conductivity with decreasing temperature. Inset: the conductivity shown over a wide energy range. (**b**) The temperature dependence of the optical conductivity for light polarized along the *b* axis. As the temperature is reduced the low-frequency conductivity decreases dramatically revealing a step-like feature at $\simeq 600$ cm^−1^; three narrow infrared-active lattice modes all lie below this energy. There is a clear transfer of spectral weight (area under the conductivity curve) from low to high frequency with decreasing temperature. The points on the conductivity axis correspond the values for *σ*_*dc*_ measured along this direction in a sample without a MIT and normalized to the extrapolated value for *σ*_1_(*ω* → 0) at room temperature. Inset: the conductivity shown over a much larger energy range. (**c**) The temperature dependence of the optical conductivity along the *c* axis, which is similar in magnitude to the conductivity along the *a* axis; a single sharp lattice mode is observed in this polarization. Inset: the conductivity shown over a wide energy range.

While the reflectance is a tremendously useful quantity, it is a combination of the real and imaginary parts of the dielectric function, and as such it is not necessarily intuitive or easily understood. It is much simpler to examine the real part of the optical conductivity, determined from a Kramers-Kronig analysis of the reflectance^19^, shown in the infrared region along the *a*, *b*, and *c* axes Figs. 2(a), (b), and (c), respectively; the insets show the conductivity over a much wider frequency range. Interestingly, the temperature dependence of the reflectance for crystals with and without an MIT is identical in the infrared region (shown for light polarized along the *b* axis in Supplementary Fig. S2). Consequently, the low-frequency optical conductivity in Fig. 2 never shows the initial increase with decreasing temperature that is seen in the dc conductivity in samples with a MIT; instead, the low-frequency optical conductivity decreases with temperature along all three lattice directions, suggesting that no MIT is present. The apparent dichotomy between the temperature dependence of the dc resistivity and the optical conductivity in crystals with an MIT [Figs. 1(b) and S2(a)] indicates that the dc transport properties are being driven by an impurity band that is sufficiently narrow so that its response falls below our lowest measured frequency.

**Figure 3:**
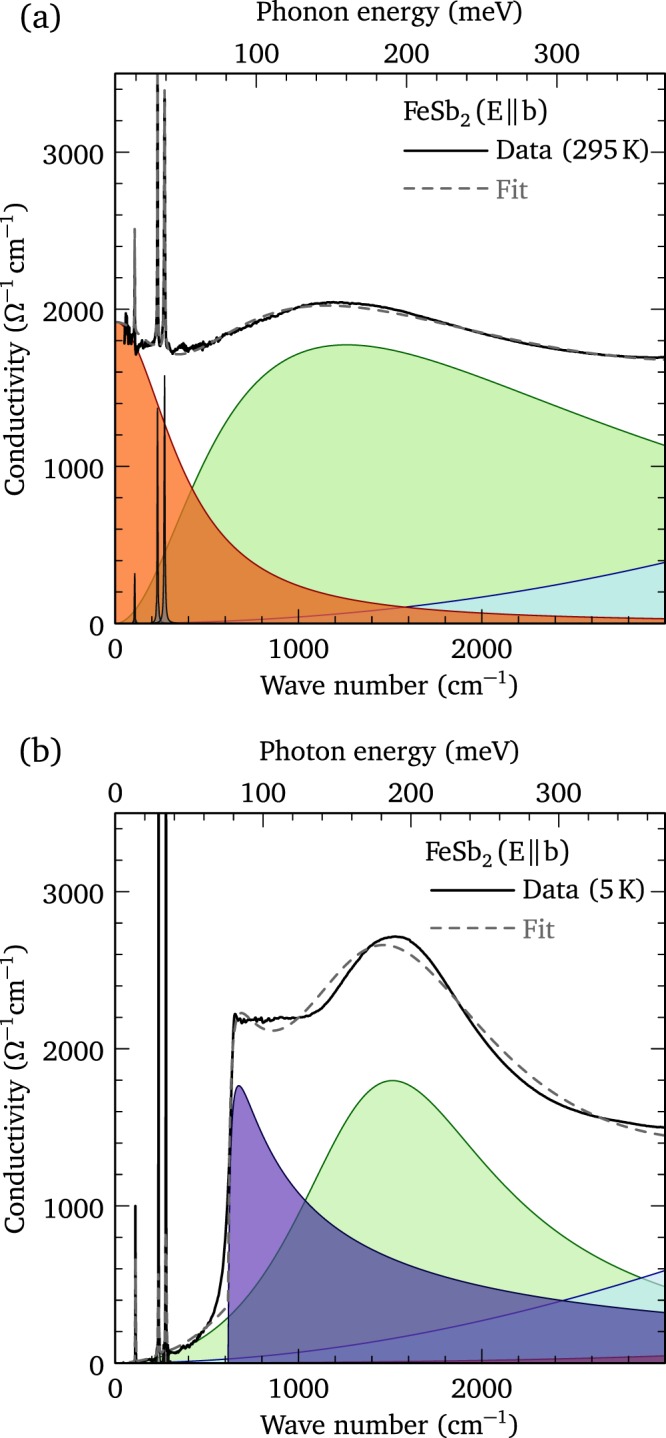
Singular behavior in FeSb_2_. (**a**) The fit to the real part of the optical conductivity of FeSb_2_ along the *b* axis at 295 K using a Drude (free carrier) component; Lorentzian oscillators with Fano profiles have been used to describe the lattice modes, while the interband (bound) excitations are assumed to be symmetric. (**b**) The fit to the gapped optical conductivity of FeSb_2_ along the *b* axis at 5 K using Fano-shaped Lorentzian oscillators to describe the lattice modes, and symmetric profiles for the interband (bound) excitations, in linear combination with the optical conductivity expected for a one-dimensional semiconductor with a characteristic $\mathrm{1/}\sqrt{w}$ singularity above the semiconducting optical gap 2Δ.

At room temperature, the real part of the optical conductivity may be described by a simple Drude model with Fano-shaped Lorentz oscillators to describe possible electron-phonon coupling^20^,

1 $${\sigma }_{1}(\omega )=\frac{2\pi }{{Z}_{0}}[\frac{{\omega }_{p}^{2}\tau }{(1+{\omega }^{2}{\tau }^{2})}+\sum _{j}\frac{{{\rm{\Omega }}}_{j}^{2}[{\gamma }_{j}{\omega }^{2}-2({\omega }^{2}{\omega }_{j}-{\omega }_{j}^{3})/{q}_{j}-{\gamma }_{j}{\omega }_{j}^{2}/{q}_{j}^{2}]}{{({\omega }^{2}-{\omega }_{j}^{2})}^{2}+{\gamma }_{j}^{2}{\omega }^{2}}],$$

where the first term denotes the (Drude) free carriers, with the square of the plasma frequency ${\omega }_{p}^{2}=4\pi n{e}^{2}/{m}^{\ast }$ and scattering rate 1/*τ*, where *n* and m^*^ are the carrier concentration and effective mass, respectively. The second term is a summation of oscillators with position *ω*_*j*_, width *γ*_*j*_, strength Ω_*j*_, and (dimensionless) asymmetry parameter $1/{q}_{j}^{2}$, that describe the vibrations of the lattice or bound excitations (interband transitions); ${Z}_{0}\simeq 377$ Ω is the impedance of free space, yielding units for the conductivity of Ω^−1^ cm^−1^. In the 1/*q*^2^ → 0 limit a symmetric Lorentzian profile is recovered; however, as 1/*q*^2^ increases the line shape becomes increasingly asymmetric. The real part of the optical conductivity along the *a* and *c* axes at 295 K, shown in Figs. 2(a) and (c), respectively, are similar, with ${\sigma }_{dc}\equiv {\sigma }_{1}(\omega \to 0)\simeq 1400$ Ω^−1^ cm^−1^. Along the *b* axis the optical conductivity at room temperature is higher, with ${\sigma }_{dc}\simeq 1900$ Ω^−1^ cm^−1^ [Fig. 2(b)]. Fits to the optical conductivity at 295 K describe the data quite well and yield ${\omega }_{p}\simeq 7690$, 6770 and 6480 cm^−1^, and $\mathrm{1/}\tau \simeq 800$, 400 and 490 cm^−1^ along the *a*, *b*, and *c* axes, respectively (Supplementary Fig. S3); the optical conductivity along the *b* axis at room temperature is shown in Fig. 3(a). This anisotropy suggests that m^*^ is slightly lower along the *a* axis, and that the larger value for *σ*_*dc*_ along the *b* axis is a consequence of a lower scattering rate (Supplementary Table 1). As Fig. 2 indicates, the Drude component begins to decrease rapidly in strength below room temperature, along all three directions, with a commensurate loss of spectral weight (the area under the conductivity curve) that is transferred from low to high frequency^6^. The Drude model may be used to track the temperature dependence of *ω*_*p*_ and 1/*τ* down to about 75 K, below which the free-carrier response becomes too small to observe in our measurements. The Drude expression for the dc conductivity, ${\sigma }_{dc}=2\pi {\omega }_{p}^{2}\tau /{Z}_{0}$, decreases rapidly as the temperature is lowered, suggesting that the transport may be described by an activation energy *E*_*a*_ using the Arrhenius equation,

2 $${\sigma }_{dc}\propto {\omega }_{p}^{2}\tau =A{e}^{-{E}_{a}/({k}_{{\rm{B}}}T)},$$

where *E*_*a*_ = *E*_*g*_/2. Transport measurements typically identify two gaps in FeSb_2_, ${E}_{g}\simeq 5$ meV below about 20 K, and ${E}_{g}\simeq 26-36$ meV in the 50−100 K temperature range^1–4^. The Arrhenius relation describes the temperature dependence of *σ*_*dc*_ along all three lattice directions quite well (see Supplementary Fig. S4), and yields values for the transport gap of ${E}_{g}\simeq 20.6$, 19.5 and 24.8 ± 2 meV along the *a*, *b*, and *c* axes, respectively, in good agreement with the high-temperature values for the transport gap.

## Dis**c**ussion

While the Drude model with Fano-shaped Lorentz oscillators is able to reproduce the temperature-dependence of the optical conductivity along the *a* and *c* axes reasonably well, it fails to describe the sharp feature that develops along the *b* axis at low temperature. This step-like feature is the signature of a van Hove singularity in the density of states. The asymmetric profile in the real part of the low-temperature optical conductivity resembles the $\mathrm{1/}\sqrt{\omega }$ singularity response observed in one-dimensional semiconductors,

3 $${\sigma }_{1{\rm{D}}}(\omega )={\sigma }_{0}[\frac{\sqrt{{\tilde{\omega }}^{2}-1}}{({\tilde{\omega }}^{2}-1)+{\xi }^{2}{\sin }^{2}\gamma }]$$

where *ξ* = *β*^2^/(1 − *β*^2^), $\gamma =\pi [\mathrm{1/2}{\beta }^{2}-1]$, and $\tilde{\omega }=\omega /2{\rm{\Delta }}$ where 2Δ is the semiconducting optical gap, and *β* is the sine-Gordon coupling constant^21^. When this functional form is taken in linear combination with several Lorentzian oscillators, the optical conductivity is reproduced quite well with *σ*_0_ = 1730 Ω^−1^ cm^−1^, 2Δ = 614 cm^−1^, and *β* = 0.75, as shown in Fig. 3(b), clearly establishing the one-dimensional nature of the optical properties. The estimate for 2Δ along the *b* axis considerably larger than *E*_*g*_; however, it should be noted that the optical determination of 2Δ probes only direct transitions between bands due to low momentum transfer. If the material has a direct gap, then the optical and transport gaps should be similar, ${E}_{g}\simeq 2{\rm{\Delta }}$; however, in indirect-gap semiconductors, phonon-assisted transitions typically result in ${E}_{g} < 2{\rm{\Delta }}$.

**Figure 4:**
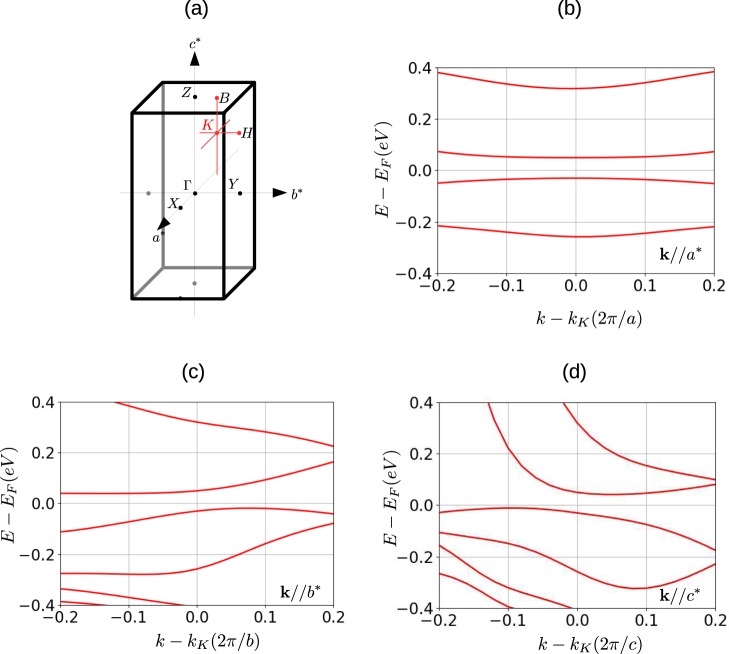
Spectral function of FeSb_2_. (**a**) First Brillioun zone and high-symmetry points of FeSb_2_ for the orthorhombic phase. Quasiparticle band structure near the K point (0.26b^*^ + 0.28c^*^) along the (**b**) a^*^ direction; (**c**) b^*^ direction; (**d**) c^*^ direction. The Brillouin zone cuts are indicated by the red lines in **a**.

The observation of one-dimensional behavior in this material is of particular importance as it has been argued that lowered dimensionality may increase the value of the Seebeck coefficient^22–24^. Electronic structure calculations can provide insight into the optical properties of a material. However, density functional theory (DFT) predicts a metallic rather than a semiconducting ground state^12^, indicating that a more sophisticated approach is required. Consequently, first principle calculations have been performed using a linearized quasiparticle self-consistent GW and dynamical mean field theory (LQSGW + DMFT) approach^25–27^ (details are provided in the Supplementary Information). Figure 4 shows the low-energy quasiparticle band structure near the K point (0.26b^*^ + 0.28c^*^) where the direct bandgap is a minimum. Here b^*^ and c^*^ are the reciprocal lattice vectors along the *b* and *c* axes. Around the K point, the calculation shows direct bandgap of $\simeq 80$ meV, which is in a good agreement with the semiconducting optical gap of $2{\rm{\Delta }}\simeq 76$ meV. In addition, low-dimensional behavior is observed near the K point; along the a^*^ direction, quasiparticle bands for the conduction and valence electrons are almost flat, as illustrated by the quasiparticle band in Fig. 4(b). In contrast, the quasiparticle bands are dispersive along the b^*^ and c^*^ directions shown in Figs. 4(c) and (d). The fact that DMFT is necessary to generate a low-dimensional quasiparticle spectral function that is consistent with the semiconducting ground state indicates that electronic correlations are an essential ingredient in understanding the anisotropic optical and transport properties of FeSb_2_.

**Figure 5:**
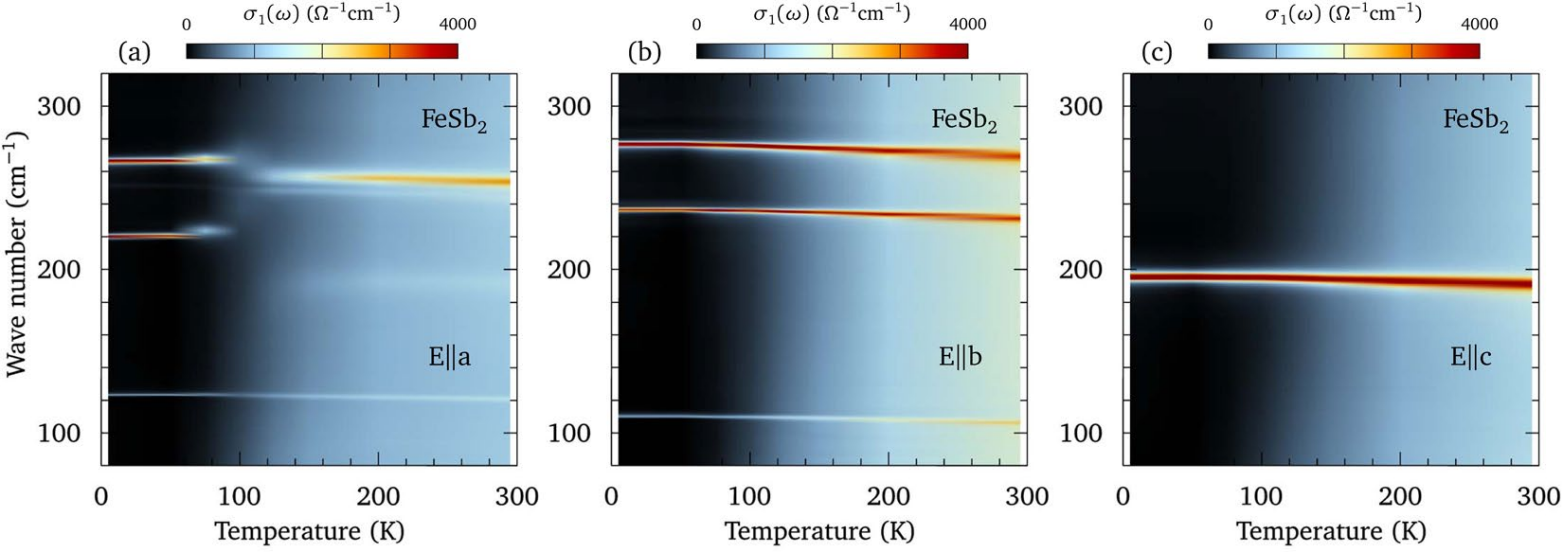
Infrared-active phonons in FeSb_2_. (**a**) The temperature-dependence of the real part of the optical conductivity for light polarized along the *a* axis projected onto the wave number versus temperature plane; the color scheme for the conductivity is shown above the plot. Only three *B*_3*u*_ modes are predicted for this symmetry; however, there are four modes at 121, 191, 243 and 254 cm^−1^ at 295 K. Below about 100 K the 191 cm^−1^ mode disappears and is replaced by a new, very strong mode at 220 cm^−1^; all the modes are quite narrow at low temperature (Table 1). The change in the character of the lattice modes below 100 K hints at a weak structural distortion along this direction. (**b**) The optical conductivity for light polarized along the *b* axis projected onto the wave number versus temperature plane. There are three strong *B*_2*u*_ modes at 106, 231, and 269 cm^−1^ at 295 K that harden and narrow while increasingly slightly in strength at low temperature. (**c**) The optical conductivity for light polarized along the *c* axis projected onto the wave number versus temperature plane. There is one strong *B*_1*u*_ mode at 191 cm^−1^ at 295 K that hardens with decreasing temperature, increasing slightly in strength and narrowing dramatically at low temperature.

We now turn our attention to the equally interesting behavior of the infrared-active lattice modes. FeSb_2_ crystallizes in the orthorhombic *Pnnm* space group, where *c* is the short axis [Fig. 1(a)]. The irreducible vibrational representation is then ${{\rm{\Gamma }}}_{irr}=2{A}_{g}+2{B}_{1g}+{B}_{2g}+{B}_{3g}+2{A}_{u}+{B}_{1u}+3{B}_{2u}+3{B}_{3u}$, of which only the *B*_1*u*_, *B*_2*u*_ and *B*_3*u*_ modes are infrared-active along the *c*, *b*, and *a* axes, respectively^6^. The temperature dependence of the real part of the optical conductivity has been projected onto the wave number versus temperature plane using the indicated color scales in Figs. 5(a), (b), and (c) for light polarized along the *a*, *b*, and *c* axes, respectively. The vibrations have been fit using oscillators with a Fano profile superimposed on an electronic background at 295 and 5 K (Supplementary Figs. S5, S6, and S7). The frequencies of the lattice modes at the center of the Brillouin zone and their atomic characters have also been calculated using first principles techniques and are in good agreement with previous results^28,29^ (details are provided in the Supplementary Information); the comparison between theory and experiment is shown in Table 1.

**Table 1:** The experimentally-observed position (*ω*_*j*_), width (*γ*_*j*_) and strength (Ω_*j*_) of the infrared-active lattice modes in FeSb_2_ along the *a* (*B*_3*u*_), *b* (*B*_2*u*_), and *c* (*B*_1*u*_) axes at 295 and 5 K, compared with the frequencies and atomic intensities calculated from first principles assuming a *Pnnm* (orthorhombic) space group; for all of the modes the asymmetry parameter $\mathrm{1/}{q}_{j}^{2}\lesssim 0.01$ (symmetric profiles). The phonon lifetimes *τ*_*j*_ ∝ 1/*γ*_*j*_. The uncertainties for the fitted position, width, and strength are estimated to be 1%, 5%, and 10%, respectively. All units are in cm^−1^, unless otherwise indicated.

Mode	Theory			Experiment					
	*ω*_*calc*_	Character		295 K			5 K		
		Fe	Sb	*ω*_*j*_	*γ*_*j*_	Ω_*j*_	*ω*_*j*_	*γ*_*j*_	Ω*j*
*B*_1 *u*_	198	0.81	0.19	191.0	4.2	1030	195.1	1.0	1120
*B*_2 *u*_	112	0.09	0.91	106.3	2.0	285	110.2	0.9	267
*B*_2 *u*_	234	0.91	0.09	231.0	3.4	608	236.5	1.0	720
*B*_2 *u*_	284	0.81	0.19	269.1	5.2	723	276.9	1.3	900
*B*_3 *u*_	125	0.10	0.90	120.8	2.1	203	123.4	0.8	229
				191.3	12.9	355			
							220.1	0.8	672
*B*_3 *u*_	252	0.98	0.02	242.9	6.1	289	251.2	3.0	130
*B*_3 *u*_	260	0.74	0.26	253.7	5.6	743	266.4	1.3	796

The behavior of the lattice modes are remarkable in several ways. Along the *a*, *b,* and *c* axes the vibrations have line widths that are up to an order of magnitude smaller than the previously reported values^6,7^; at low temperature all the modes are extremely sharp and several have line widths of less than 1 cm^−1^, a result that has also been observed in some Raman-active modes^30^. The narrow line widths imply long phonon lifetimes (${\tau }_{j}\propto \mathrm{1/}{\gamma }_{j}$) and mean-free paths, consistent with the suggestion of quasi-ballistic phonons^11,15^, which affect *S* through the phonon-drag effect where the phonon current drags the charge carriers, giving rise to an additional thermoelectric voltage^31–33^. In addition, while several of the infrared-active vibrations were previously reported to have a slightly asymmetric profile at high temperature^6,7^, in this work all the line shapes appear to be symmetric ($1/{q}_{j}^{2}\simeq 0$), indicating that electron-phonon coupling is either very weak or totally absent. The single *B*_1*u*_ mode along the *c* axis, and the three *B*_2*u*_ modes along the *b* axis, shown in Figs. 5(c) and (b), respectively, increase in frequency (harden) with decreasing temperature, and are in excellent agreement with the calculated values (Table 1). The behavior of the lattice modes along the *a* axis in Fig. 5(a) are dramatically different. At room temperature the three modes observed at $\simeq 121$, 243, and 254 cm^−1^ are in good agreement well with the calculated values for the *B*_3*u*_ modes at 125, 252, and 260 cm^−1^, respectively; however, a fourth reasonably strong mode at 191 cm^−1^ is also observed that is considerably broader than the other vibrations. As the temperature is reduced the mode at 191 cm^−1^ actually decreases slightly in frequency, while the remaining modes harden. Below about 100 K, the mode at $\simeq 191$ cm^−1^ vanishes and a new, very strong mode appears at $\simeq 220$ cm^−1^, while at the same time the modes at 243 and 254 cm^−1^ both shift to slightly higher frequencies; the mode at 121 cm^−1^ shows no signs of any anomalous behavior [Fig. 5(a) and Fig. S5]. The fate of the 191 cm^−1^ mode is uncertain; however, it is unlikely that it has evolved into the 220 cm^−1^ mode due to the large difference in oscillator strengths (Table 1). It is also unlikely that this is a manifestation of the *B*_1*u*_ mode, which has a comparable frequency, because that feature does not display the unusual temperature dependence of the mode observed along the *a* axis, nor is there any evidence of it along the *b* axis. The dramatic change in the nature of the lattice modes along the *a* axis at precisely the temperature where the resistivity begins to increase dramatically suggests there is a weak structural distortion or phase transition.

To conclude, the temperature dependence of the optical and dc transport properties of single crystals of FeSb_2_, both with and without a MIT, have been examined over a wide temperature and spectral range, along all three lattice directions. While the temperature dependence of the optical properties are essentially identical in the two types of crystals, the dc transport properties are dramatically different. This dichotomy can be explained by the presence of a sample-dependent impurity band that lies below the optical measurements. The optical conductivity in both types of crystals reveals an anisotropic response at room temperature, and singular behavior at low temperature along the *b* axis, demonstrating a one-dimensional semiconducting response with $2{\rm{\Delta }}\simeq 76$ meV, in agreement with *ab inito* calculations. The lattice modes along the *b* and *c* axes have symmetric profiles which narrow and harden with decreasing temperature, and their positions are in good agreement with first principles calculations. However, along the *a* axis there is an extra mode above 100 K; below this temperature the resistivity increases rapidly and the high-frequency vibrational modes undergo significant changes that hint a weak structural distortion or transition. Transport studies along this direction may shed light on the nature of this peculiar behavior. Although electron-phonon coupling is apparently either very weak or totally absent in this material, the fact that DMFT is required to reproduce the semiconducting ground state and anisotropic response indicates that electronic correlations play an important role in the optical and transport properties. While the extremely narrow phonon line shapes support the phonon-drag explanation of the high thermoelectric power, electronic correlations and the low-dimensional behavior along the *b* axis may also enhance the Seebeck coefficient^22–24^, making it likely that both contribute to the extremely high thermopower observed in FeSb_2_.

## Methods

The temperature dependence of the absolute reflectance was measured at a near-normal angle of incidence using an *in situ* evaporation method^18^ over a wide frequency range on Bruker IFS 113v and Vertex 80v spectrometers. In this study mirror-like as-grown faces of single crystals have been examined. After an initial measurement, the *c*-axis face was determined to have a minor surface irregularity, so it was was polished and remeasured. Polishing broadens the lattice mode somewhat, but the electronic properties were not affected. The temperature dependence of the reflectance was measured up to $\simeq 1.5$ eV, while polarization studies were conducted up to at least 3 eV. The complex optical properties were determined from a Kramers-Kronig analysis of the reflectance^19^. The Kramers-Kronig transform requires that the reflectance be determined for all frequencies, thus extrapolations must be supplied in the *ω* → 0,∞ limits. In the metallic state the low frequency extrapolation follows the Hagen-Rubens form, $R(\omega )\propto 1-\sqrt{\omega }$, while in the semiconducting state the reflectance was continued smoothly from the lowest measured frequency point to $R(\omega \to \mathrm{0)}\simeq 0.64$ and 0.68 along the *a* and *c* axes, respectively, and $\simeq 0.74$ along the *b* axis. The reflectance is assumed to be constant above the highest measured frequency point up to $\simeq 8\times {10}^{4}$ cm^−1^, above which a free electron gas asymptotic reflectance extrapolation *R*(*ω*) ∝ 1/*ω*^4^ is employed^34^.

## Electronic supplementary material

Supplementary Information
