# Supplementary material for: Unusual electronic and vibrational properties in the colossal thermopower material FeSb2
Source: Sci Rep. 2018 Aug 3;8:11692. doi: 10.1038/s41598-018-29909-2 (PMC6076321; doi:10.1038/s41598-018-29909-2)
Supplement: Supplementary file 1 — Supplementary Information [file 41598_2018_29909_MOESM1_ESM.pdf]

# Supplementary Information

# Unusual electronic and vibrational properties in the colossal thermopower material $FeSb_2$

C. C. Homes[1,*], Q. Du[1,2], C. Petrovic[1,2], W. H. Brito[1], S. Choi[1], & G. Kotliar[1,3]

[1] *Condensed Matter Physics and Materials Science Division, Brookhaven National Laboratory, Upton, New York 11973, USA*

[2] *Department of Materials Science and Chemical Engineering, Stony Brook University, Stony Brook New York 11790, USA*

[3] *Department of Physics and Astronomy, Rutgers, The State University of New Jersey, Piscataway, New Jersey 08854, USA*

[*] Corresponding author: homes@bnl.gov.

**Reflectance.** The reflectance has been measured along the *a*, *b*, and *c* axes at a near-normal angle of incidence over a wide temperature and frequency range using an overfilling technique[1], shown in Figs. S1(a), (b), and (c), respectively. At room temperature, the reflectance is characteristic of a poor metal along all three lattice directions, although along the *b* axis the reflectance is noticeably higher than the other two directions, indicating an anisotropic response. The infrared-active lattice modes are only weakly visible at high temperature; however, as the temperature is lowered the low-frequency reflectance decreases, signaling an increasingly insulating response and the sharp, unscreened lattice modes come to dominate the infrared reflectance. Interestingly, a sharp upturn may still be observed in the reflectance at low-frequency even at $\simeq$ 50 to 75 K, indicating that

free carriers are still present [in a metallic system, $R(\omega \to 0) = 1$]. Along the *a* axis there is a significant change in the vibrational properties below about 100 K, suggesting a weak structural distortion or transition. While this behavior is not observed along the *b* axis, an unusual cusp-like feature at about 600 cm$^{-1}$ develops at low temperature. Finally, the temperature dependence along the *c* axis is similar to that observed along *a*; however, only one strong lattice mode is observed in this direction at low temperature.

**Principle optical axes.** Samples with a metal-insulator transition (MIT) are observed to grow with well defined rectangular *a*-*b* crystal faces with good, optical-quality surfaces. The long growth direction is the *b* axis, and the perpendicular direction is the *a* axis (verified using x-ray diffraction). Samples without a MIT still possess mirror-like optical surfaces, but there are no clear growth directions or crystal edges suitable for determining an orientation. In samples with a MIT, at low temperature it was noted that along the *a* axis the low-frequency $B_{3u}$ mode is located at $\simeq$ 123 cm$^{-1}$, and along the *b* axis the low-frequency $B_{2u}$ mode is about 13 cm$^{-1}$ lower at $\simeq 110$ cm$^{-1}$. In samples without a MIT, an *a*-*b* face could be isolated and then the polarization dependence of the low-frequency mode in the reflectance studied at low-temperature. By determining those polarization where only the $B_{2u}$ or $B_{3u}$ low-frequency mode is active, it is possible to determine the directions of the *a* and *b* axes; using different polarizers but the same orientation, the polarization dependence of the reflectance may then be examined over a wide spectral region. In both types of crystals, the *c* axis may also be determined in this manner by selecting the polarization in which only the strong $B_{1u}$ phonon is active.

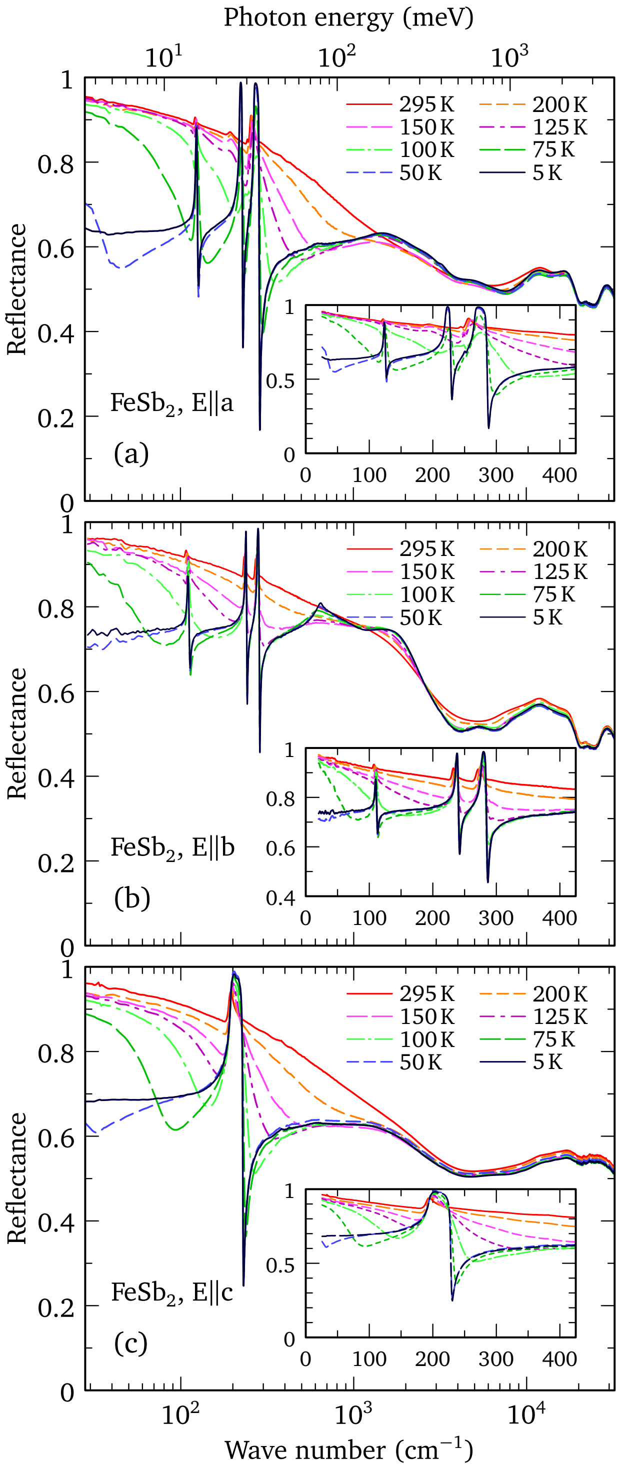


**Figure S1 | Reflectance of $FeSb_2$.**

**a**, The temperature dependence of the reflectance over a wide frequency range for light polarized along the *a* axis. Inset: The reflectance shown in the low-frequency region, revealing that below

about 100 K there is a dramatic change in the nature of the lattice modes. **b**, The reflectance for light polarized along the $b$ axis, showing the emergence of an unusual cusp-like feature at $\simeq 600$ cm$^{-1}$ at low temperature. Inset: The reflectance shown in the low-frequency region. **c**, The reflectance for light polarized along the $c$ axis. Inset: The low-frequency reflectance showing the emergence of the single strong lattice mode.

**Reflectance of samples with and without a MIT.** The reflectance of crystals with and without a MIT has been measured. The temperature dependence the reflectance of a single crystal of $FeSb_2$ with a MIT (R046) for light polarized along the $b$ axis is shown in Fig. S2(a); opposite this figure is the temperature dependence of the reflectance of a single crystal of $FeSb_2$ without a MIT (R472).

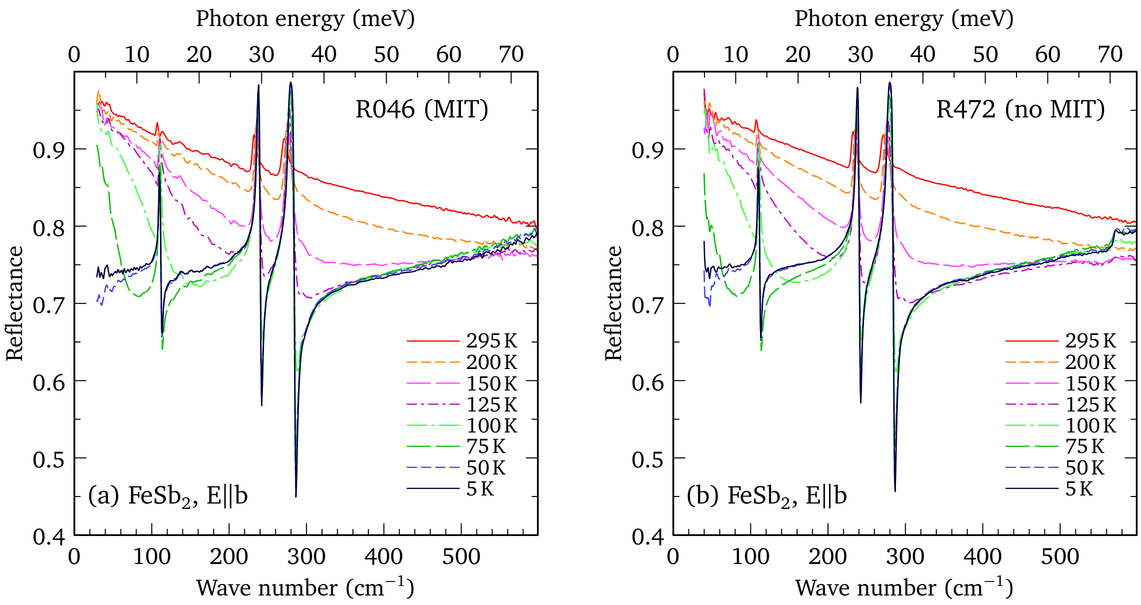


**Figure S2 | Infrared reflectance in materials with and without a MIT.**

The temperature dependence of the reflectance for light polarized along the $b$ axis in crystals (a) with (R046), and (b) without (R472) a MIT.

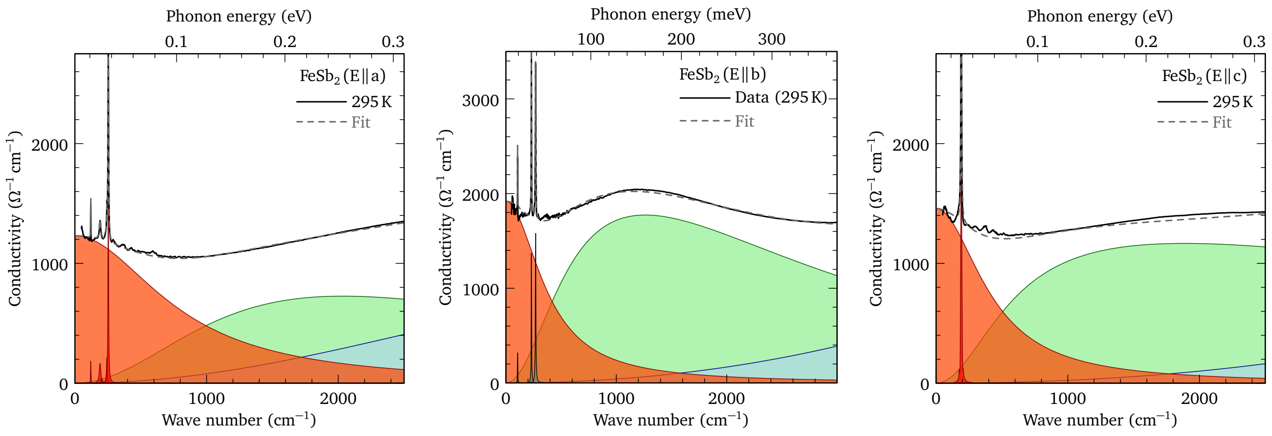


**Figure S3 | Drude-Lorentz fits.**

The Drude-Lorentz fits to the real part of the optical conductivity in $FeSb_2$ at 295 K along the $a$, $b$, and $c$ axis, respectively, where the individual Drude and Lorentz contributions are shown (see Table 1). The sharp features are the infrared-active lattice modes.

for light polarized along the *b* axis, Fig. S2(b). A casual inspection reveals that despite the different resistivity curves in Fig. 1(b) in the main text, the reflectance curves for the two crystals are essentially identical.

**Drude-Lorentz model.** At room temperature the Drude-Lorentz model for the dielectric function describes the optical conductivity quite well,

$$\tilde{\epsilon}(\omega) = \epsilon_\infty - \frac{\omega_{p,D}^2}{\omega^2 + i\omega/\tau_D} + \sum_j \frac{\Omega_j^2}{\omega_j^2 - \omega^2 - i\omega\gamma_j}, \quad (1)$$

where $\epsilon_\infty$ high-frequency contribution to the real part of the dielectric function. In the first term $\omega_{p,D}^2 = 4\pi n e^2/m^*$ and $1/\tau_D$ are the square of the plasma frequency and scattering rate for the

delocalized (Drude) carriers, respectively, and $n$ and $m^*$ are the carrier concentration and effective mass. In the summation, $\omega_j$, $\gamma_j$ and $\Omega_j$ are the position, width, and strength of a symmetric Lorentzian oscillator that describe the $j$th vibration or bound excitation. The complex conductivity is $\tilde{\sigma}(\omega) = \sigma_1 + i\sigma_2 = -2\pi i\omega[\tilde{\epsilon}(\omega) - \epsilon_\infty]/Z_0$ (in units of $\Omega^{-1}$cm$^{-1}$), where $Z_0 \simeq 377\ \Omega$ is the impedance of free space. The real part of the optical conductivity is given by

$$\sigma_1(\omega) = \frac{2\pi}{Z_0}\left[\frac{\omega_p^2\,\tau}{(1+\omega^2\tau^2)} + \sum_j \frac{\omega^2\gamma_j\Omega_j^2}{(\omega_j^2-\omega^2)^2+\gamma_j^2\omega^2}\right]. \qquad (2)$$

Fits to the real part of the optical conductivity along the $a$, $b$, and $c$ axis at 295 K are shown in Fig. S3. The Drude component may be tracked down to roughly 75 K. The Drude parameters determined from the fits are listed in Table 1.

**Activation energies.** The Drude expression for the dc conductivity, $\sigma_{dc} = 2\pi\omega_p^2\tau/Z_0$, decreases rapidly below room temperature along all three lattice directions, as indicated in Table 1. This suggests that the transport may be described by the Arrhenius equation $\sigma_{dc} \propto \omega_p^2\tau = A\,e^{-E_a/(k_BT)}$, or $\ln(\sigma_{dc}) = -E_a/(k_BT)$, where the activation energy $E_a = E_g/2$. Using the temperature dependence of $\sigma_{dc}$ determined from the Drude parameters along the three lattice directions in Table 1, it is possible to estimate transport gaps of $E_g$ = 20.6, 19.5, and 24.8$\pm$2 meV from linear regressions, shown in Fig. S4.

**Infrared-active vibrational modes.** The Lorentz oscillators discussed in the previous section display a symmetric profile; however, in previous optical studies it was argued that several of the infrared-active lattice modes had asymmetric line shapes, which was taken as evidence of

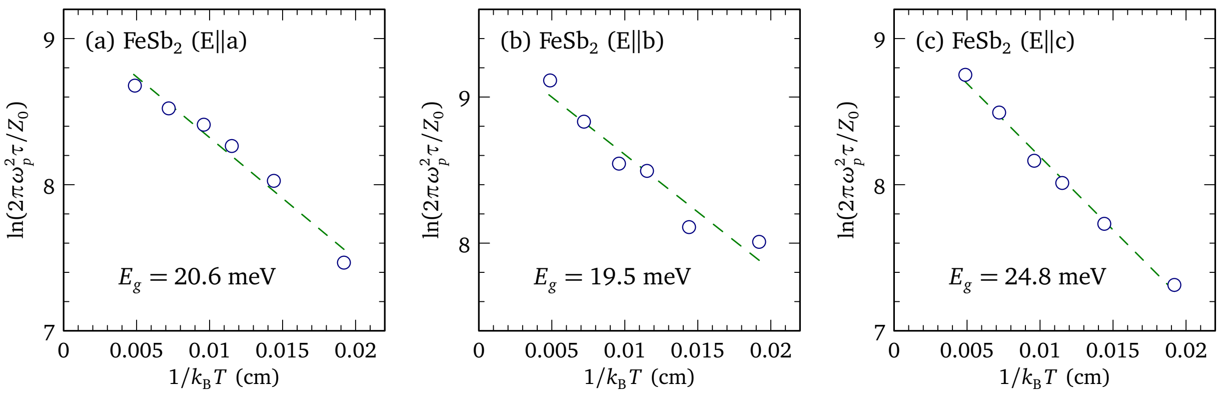


**Figure S4 | Activation energies.**

The estimate of the transport gap $E_g$ from the natural log of the Drude expression of the dc conductivity, $\ln{(2\pi\omega_p^2\tau/Z_0)}$, in $FeSb_2$ versus $1/k_{\rm B}T$ (Table 1) and the results of the linear regression (dashed line) along (a) the *a* axis, with $E_g = 20.6 \pm 2$ meV; (b) the *b* axis, with $E_g = 19.5 \pm 2$ meV; (c) the *c* axis, with $E_g = 24.8 \pm 2$ meV.

electron-phonon coupling[2,3]. Accordingly, we have fit the infrared-active lattice modes using an asymmetric Fano profile. The real and imaginary parts of the optical conductivity are[4]

$$\sigma_1(\omega) = \frac{2\pi}{Z_0}\frac{\Omega_0^2\left[\gamma_0\omega^2 - 2(\omega^2\omega_0 - \omega_0^3)/q_0 - \gamma_0\omega_0^2/q_0^2\right]}{(\omega^2-\omega_0^2)^2 + \gamma_0^2\omega^2}, \tag{3}$$

and

$$\sigma_2(\omega) = \frac{2\pi}{Z_0}\frac{\omega\Omega_0^2\left[(\omega^2 - \omega_0^2) - 2\gamma_0\omega_0/q_0 + (\omega^2 - \omega_0^2 + \gamma_0^2)/q_0^2\right]}{(\omega^2-\omega_0^2)^2 + \gamma_0^2\omega^2}, \tag{4}$$

where $\omega_0$, $\gamma_0$ and $\Omega_0$ are the position, width, and strength of the vibration, respectively, and the asymmetry is described by the dimensionless parameter $1/q_0 = \omega_q/\omega_0$. Note that for finite $\omega_0$, in the $\omega_q \rightarrow 0$ (or $1/q_0^2 \rightarrow 0$) limit, a Lorentz oscillator is recovered; however, as $1/q_0^2$ increases the line shape becomes increasingly asymmetric. The oscillators are superimposed on an electronic

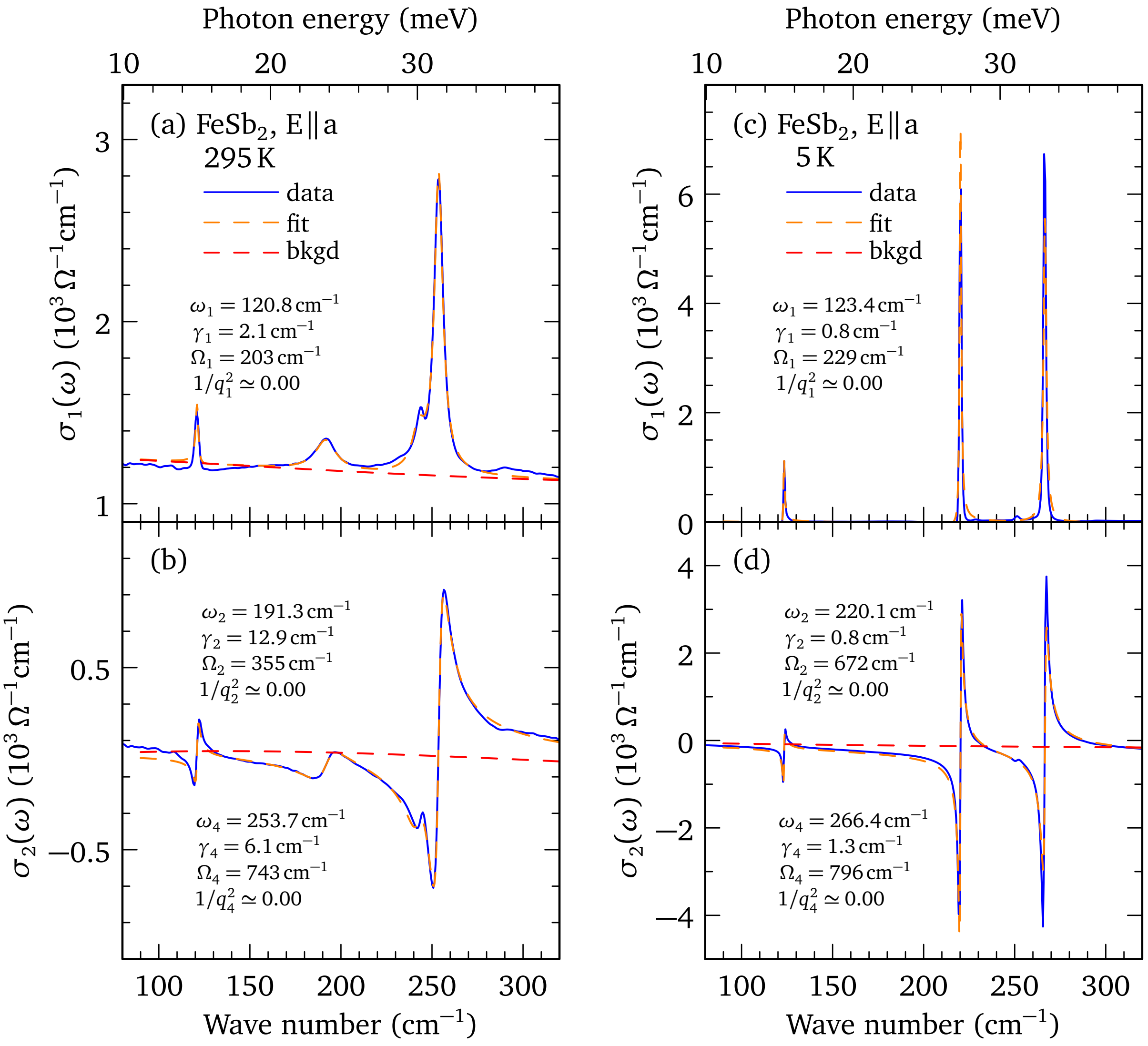


**Figure S5** | **Fano fits along the $a$ axis.**

The Fano fits to the (a) real and (b) imaginary parts of the optical conductivity of $FeSb_2$ at 295 K for light polarized along the $a$ axis, and the same fits for the (c) real and (d) imaginary parts at 5 K. Note the dramatic change in the nature of the lattice modes at low temperature as the vibration at $\simeq$ 191 cm$^{-1}$ has been replaced by a very strong mode at $\simeq$ 220 cm$^{-1}$; all the modes narrow dramatically at low temperature. The results are summarized in Table 1 in the main text.

background that is described by the Drude-Lorentz model; the real and imaginary parts of the optical conductivity are fit simultaneously using a non-linear least-squares technique. The results of the Fano fits to the complex conductivity for light polarized along the *a* axis are shown in Fig. S5 at 295 and 5 K. At room temperature there are four modes at $\simeq$ 121, 191, 243, and 254 $cm^{-1}$ superimposed upon an electronic background, shown in Figs. S5(a) and (b); the mode at $\simeq$ 191 $cm^{-1}$ is broader than the other modes. At 5 K there are still four modes but the electronic background has been removed due to the formation of an optical gap, shown in Figs. S5(c) and (d). The mode at $\simeq$ 191 $cm^{-1}$ has been replaced by a very strong mode at $\simeq$ 220 $cm^{-1}$. All the vibrations have narrowed significantly, and are much narrower than previously reported[2,3], although it has been noted that at low temperature the Raman-active modes have comparable line widths[5]; none of these modes display any significant asymmetry.

The results of the Fano fits to the complex conductivity for light polarized along the *b* axis are shown in Fig. S6 at 295 and 5 K. At room temperature, Figs. S6(a) and (b), there are three modes superimposed upon an electronic background, of which only the low-frequency mode displays any hint of an asymmetry. At low temperature Figs. S6(c) and (d), the electronic background has been removed and the three modes are now extremely narrow; none of these modes display any asymmetry.

The results of the Fano fits to the complex conductivity for light polarized along the *c* axis are shown in Fig. S7 at 295 and 5 K for an unpolished sample, which was initially investigated in the far-infrared region, and then subsequently polished. Polishing broadens the vibrational modes by

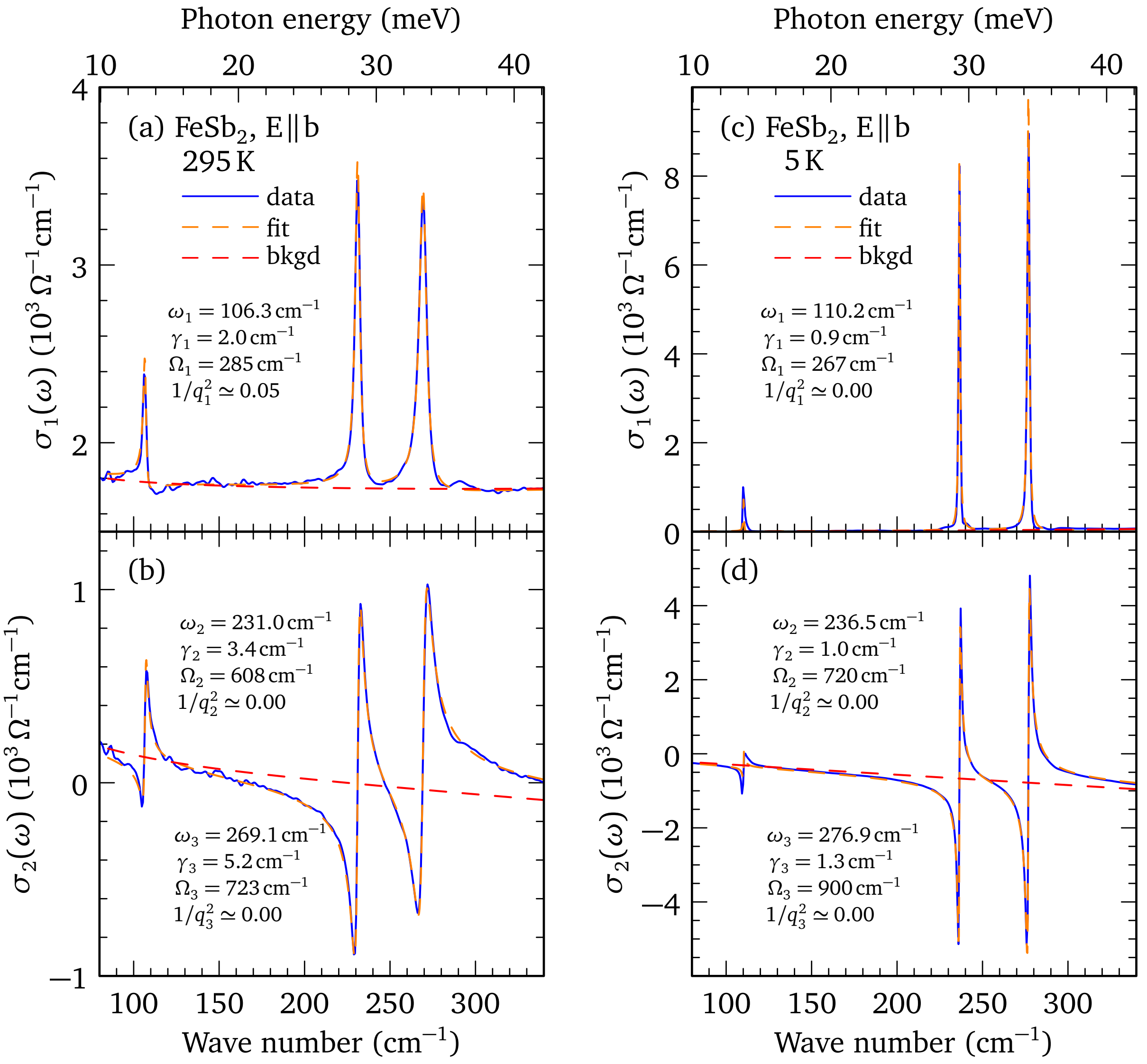


**Figure S6 | Fano fits along the *b* axis.**

The Fano fits to the (a) real and (b) imaginary parts of the optical conductivity of $FeSb_2$ at 295 K for light polarized along the *b* axis, and the same fits for the (c) real and (d) imaginary parts at 5 K. At low temperature, the modes have narrowed dramatically; however, unlike the *a* axis, there are only minor changes in the nature of the modes. The results are summarized in Table 1 in the main text.

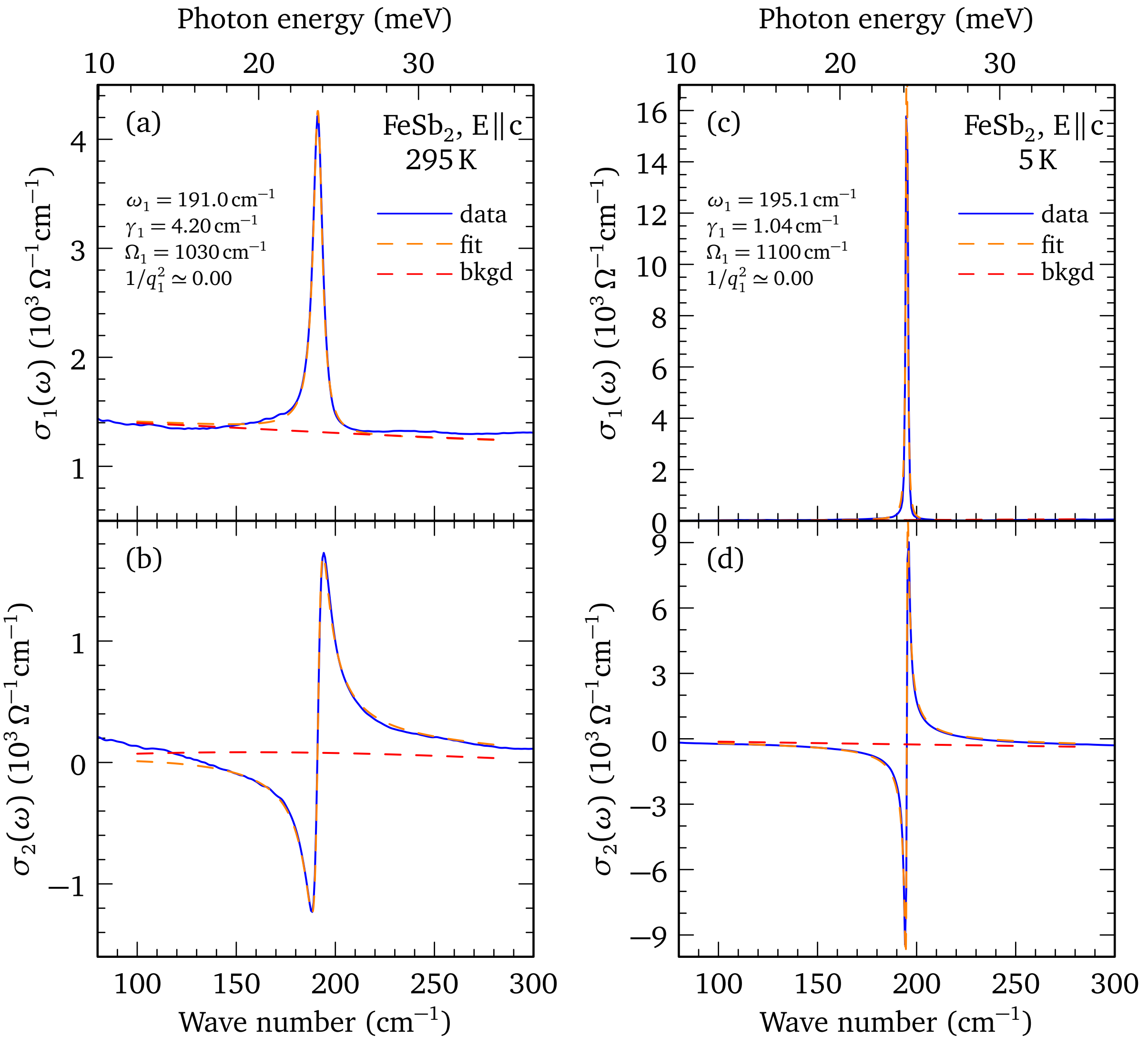


**Figure S7** | **Fano fits along the *c* axis.**

The Fano fits to the (a) real and (b) imaginary parts of the optical conductivity of (unpolished) $FeSb_2$ at 295 K for light polarized along the *c* axis, and the same fits for the (c) real and (d) imaginary parts at 5 K. There is only one strong mode active in this direction; at low temperature this mode has narrowed dramatically; however, this is little or no evidence of any asymmetry. The results are summarized in Table 1 in the main text.

about $30 - 40\%$. For the vibrational fits, the low-frequency data for the unpolished sample was merged with the high-frequency data for the polished sample in order to perform a Kramers-Kronig analysis, allowing the vibrational features to be studied in the absence of mechanical damage to the surface that result from polishing. At room temperature, Figs. S7(a) and (b), there is only one strong mode superimposed upon an electronic background. At low temperature, Figs. S7(c) and (d), the electronic background has been removed due to the formation of an optical gap and the mode is now very narrow; however, the strength has not changed appreciably. This mode displays little or no asymmetry. The results of these fits are summarized in Table 1 in the main text.

**Spectral functions.** To understand the one-dimensional nature of optical properties at low-temperature, we calculated spectral function of $FeSb_2$ in the orthorhombic phase within LQSGW+DMFT[6] using COMSUITE[7]. We do a self-consistent QSGW calculation and then calculate the local self-energy using DMFT with static $U_d$ and $J_H$ without feedback to a non-local self-energy within GW. For the DMFT step, we choose a very localized orbital spanning a large energy window, which contains the most strongly hybridized bands, as well as upper and lower Hubbard bands. For the GW calculation the FlapwMBPT code was used[8–10]. We have used the room temperature atomic positions[11] ($a = 5.83$ Å, $b = 6.54$ Å, and $c = 3.20$ Å). The following parameters for the basis set are used. Muffin-tin (MT) radii (RMT) in units of the Bohr radius are 2.6 for Fe and 2.2 for Sb. Wave functions are expanded in spherical harmonics with $l$ up to 4 for Fe and 4 for Sb in the MT spheres, and by plane waves with the energy cutoff determined by $\mathrm{RMT_{Fe}} \times \mathrm{k_{max}} = 8.1$ in the interstitial (IS) region. The Brillouin zone was sampled with $4 \times 4 \times 8$ $k$-point grid. The product basis is expanded by spherical harmonics with $l$ up to $l_{max} = 6$ in the MT spheres and

$\mathrm{RMT_{Fe}} \times \mathrm{k_{max}} = 11.4$ in IS region. Unoccupied states with an energy up to 200 eV from the Fermi energy are taken into account for the polarizability and self-energy calculations. Spin-orbit coupling was not included. Within the DMFT loop we use a Wannier-interpolated MQSGW band structure using a maximally localized Wannier function (MLWF)[12], and then constructed a local projector in a $15 \times 15 \times 15$ $k$-grid in the energy window $\mathrm{E_F} \pm 8$ eV, where $\mathrm{E_F}$ is the Fermi level. The static $U_d$ and $J_H$ associated with constructed Fe-$d$ projectors are evaluated by a modification of the constrained random phase approximation method[6,13] which avoids screening by the correlated as well as the hybridized bands. We divide the dynamic polarizability within the MQSGW approximation $\chi_{QP}$ into two parts, $\chi_{QP} = \chi_{QP}^{low} + \chi_{QP}^{high}$. Here, $\chi_{QP}^{low}$ is defined by all transitions between the states in the low-energy Hamiltonian constructed by MLWFs. Using $\chi_{QP}^{high}$, we evaluate the partially-screened Coulomb interaction $U^{-1}(r, r', k, i\omega_n) = V^{-1}(r, r', k, i\omega_n) - \chi_{QP}^{high}(r, r', k, i\omega_n)$ and parameterize the static $U_d = 4.8$ eV and $J_H = 1.0$ eV by Slater's integrals[14,15] where $V$ is bare Coulomb interaction. The Feynman graphs included in both MQSGW and DMFT (double counting) are the local Hartree and local GW diagram; they are computed using the local projection of the MQSGW Green's function and the local Coulomb matrix constructed from Slater's integrals.

**Lattice dynamics.** The total energy of $FeSb_2$ for the orthorhombic ($Pnnm$) phase was calculated using density functional theory (DFT) with the generalized gradient approximation (GGA) using the full-potential linearized augmented plane-wave (FP-LAPW) method[16] with local-orbital extensions[17] in the WIEN2k implementation[18]. An examination of different Monkhorst-Pack $k$-point meshes indicated that a $4 \times 3 \times 7$ $k$-point mesh with $R_{mt}k_{max} = 8$ was sufficient for good energy convergence. Beginning with the experimental unit cell[11], the lattice parameters are ad-

justed and the total energy calculated for each structure; the lowest total energy in this phase space corresponds to the most stable geometry. The atomic fractional coordinates were then relaxed with respect to the total force, typically resulting in residual forces of less than 0.1 mRy/a.u. per atom. This procedure was repeated until no further improvement was obtained. The relaxed unit cell parameters of 5.82, 6.52 and 3.17 Å for the $a$, $b$, and $c$ axes, respectively, are only slightly smaller than the experimentally observed values of 5.83, 6.53, and 3.20 Å; the fractional coordinates for Sb $(x, y, 0)$ with $x = 0.1895$ and $y = 0.3536$ are in good agreement with the experimental values of $x = 0.1885$ and $y = 0.3561$ [due to symmetry constraints, the fractional coordinates for the Fe atom are kept fixed at $(0, 0, 0)$]. The lattice vibrations have been determined using the direct method, also known as the frozen-phonon technique. To determine the phonons at the zone center, a $1 \times 1 \times 1$ supercell is sufficient. To obtain a complete set of Hellmann-Feynman forces, a total of 6 independent displacements are required; because there are always some residual forces at the atomic sites we have considered symmetric displacements, which doubles this number, resulting in a total of 12 atomic displacements. In this case, displacement amplitudes of 0.03 Å were used; typical values for the displacements range from 0.02 to 0.06 Å. The calculations have converged when the successive changes for the forces on each atom are less than 0.01 mRy/a.u. The residual forces are collected for each set of symmetric displacements and a list of the Hellmann-Feynman forces are generated. Using the program PHONON[19] the cumulative force constants deconvoluted from the Hellmann-Feynman forces are introduced into the dynamical matrix, which is then diagonalized in order to obtain the phonon frequencies. The atomic intensities are further calculated to describe the character of the vibration; in this case the intensity refers to the square of the

vibrational amplitude of each atom for a given mode.

Table 1: The temperature dependence of the Drude plasma frequency ($\omega_p$) and scattering rate ($1/\tau$) returned from the fit of the Drude-Lorentz model to the real part of the optical conductivity in $FeSb_2$ along the $a$, $b$, and $c$ axes. All units are in $cm^{-1}$, unless otherwise noted.

| | $E \parallel a$ | | $E \parallel b$ | | $E \parallel c$ | |
|---|---|---|---|---|---|---|
| T (K) | $\omega_p$ | $1/\tau$ | $\omega_p$ | $1/\tau$ | $\omega_p$ | $1/\tau$ |
| 295 | 7687 | 799 | 6765 | 400 | 6475 | 487 |
| 200 | 5674 | 508 | 5464 | 346 | 5085 | 420 |
| 150 | 3971 | 281 | 3643 | 205 | 3239 | 237 |
| 125 | 3087 | 195 | 2720 | 120 | 2356 | 146 |
| 100 | 2060 | 110 | 1899 | 86 | 1555 | 84 |
| 75 | 1154 | 60 | 1087 | 31 | 859 | 39 |

**References.**


1. Homes, C. C., Reedyk, M., Crandles, D. A. & Timusk, T. Technique for measuring the reflectance of irregular, submillimeter-sized samples. *Appl. Opt.* **32**, 2976–2983 (1993).

2. Perucchi, A., Degiorgi, L., Hu, Rongwei, Petrovic, C. & Mitrović, V. F. Optical investigation of the metal-insulator transition in $FeSb_2$. *Eur. Phys. J. B* **54**, 175–183 (2006).

3. Herzog, A. *et al.* Strong electron correlations in $FeSb_2$: An optical investigation and comparison with $RuSb_2$. *Phys. Rev. B* **82**, 245205 (2010).

4. Homes, C. C., Dai, Y. M., Akrap, A., Bud'ko, S. L. & Canfield, P. C. Vibrational anomalies in $A\mathrm{Fe_2As_2}$ ($A$=Ca, Sr, and Ba) single crystals. *Phys. Rev. B* **98**, 035103 (2018).

5. Lazarević, N., Popović, Z. V., Hu, R. & Petrovic, C. Evidence for electron-phonon interaction in $\mathrm{Fe}_{1-x}M_x\mathrm{Sb}_2$ ($M$ = Co and Cr; $0 \leq x \leq 0.5$) single crystals. *Phys. Rev. B* **81**, 144302 (2010).

6. Choi, S., Kutepov, A., Haule, K., van Schilfgaarde, M. & Kotliar, G. First-principles treatment of Mott insulators: linearized QSGW+DMFT approach. *Quantum Mater.* **1**, 16001 (2016).

7. https://www.bnl.gov/comscope.

8. Kutepov, A., Savrasov, S. Y. & Kotliar, G. Ground-state properties of simple elements from GW calculations. *Phys. Rev. B* **80**, 041103 (2009).

9. Kutepov, A., Haule, K., Savrasov, S. Y. & Kotliar, G. Electronic structure of Pu and Am metals by self-consistent relativistic $GW$ method. *Phys. Rev. B* **85**, 155129 (2012).

10. Kutepov, A. L., Oudovenko, V. S. & Kotliar, G. Linearized self-consistent quasiparticle GW method: Application to semiconductors and simple metals. *Comp. Phys. Commun.* **219**, 407–414 (2017).

11. Holseth, H. & Kjekshus, A. Compounds with the Marcasite Type Crystal Structure. IV. The Crystal Structure of $FeSb_2$. *Acta Chem. Scand.* **23**, 3043–3050 (1968).

12. Mostofi, A. A. *et al.* wannier90: A tool for obtaining maximally-localised Wannier functions. *Comp. Phys. Commun.* **178**, 685–699 (2008).

13. Aryasetiawan, F. *et al.* Frequency-dependent local interactions and low-energy effective models from electronic structure calculations. *Phys. Rev. B* **70**, 195104 (2004).

14. van der Marel, D. & Sawatzky, G. A. Electron-electron interaction and localization in $d$ and $f$ transition metals. *Phys. Rev. B* **37**, 10674–10684 (1988).

15. Kutepov, A., Haule, K., Savrasov, S. Y. & Kotliar, G. Self-consistent $GW$ determination of the interaction strength: Application to the iron arsenide superconductors. *Phys. Rev. B* **82**, 045105 (2010).

16. Singh, D. J. *Planewaves, Pseudopotentials and the LAPW method* (Kluwer Adademic, Boston, 1994).

17. Singh, D. Ground-state properties of lanthanum: Treatment of extended-core states. *Phys. Rev. B* **43**, 6388–6392 (1991).

18. P. Blaha, K. Schwarz, G. K. H. Madsen, D. Kvasnicka and J. Luitz, WIEN2k, *An augmented plane wave plus local orbitals program for calculating crystal properties* (Techn. Universität Wien, Austria, 2001).

19. K. Parlinksi, Software PHONON (2003).
